# Multi-Omics Insights into Altered Flavonoid Metabolism Underlying Skin Color Variation in a Bud Mutant of *Vitis vinifera* Zaoheibao

**DOI:** 10.3390/metabo15100675

**Published:** 2025-10-16

**Authors:** Liping Huang, Xi Dai, Linan Zhang, Yue Zhu, Min Wang, Zhili Xun, Qifeng Zhao, Jiancheng Zhang

**Affiliations:** 1 Pomology Institute, Shanxi Agricultural University, Jinzhong 030801, China; hlp-23@163.com (L.H.); zhy1012@sxau.edu.cn (Y.Z.); wangmin@163.com (M.W.); xzlgss@163.com (Z.X.); 2 College of Horticulture, Shanxi Agricultural University, Jinzhong 030801, China; daiqian@yy201902b.wecom.work (X.D.); 15735734695@163.com (L.Z.)

**Keywords:** grape (*Vitis vinifera*), bud sport, anthocyanin biosynthesis, transcriptome, LC-MS metabolome, MYB

## Abstract

Background: Fruit skin color is a key determinant of grape quality and market value, primarily governed by anthocyanin biosynthesis. Methods: In this study, we explored the molecular basis of skin color variation in the grape cultivar Zaoheibao and its bud mutant, which displays a striking shift from purple-black to yellow-green. Integrated metabolomic and transcriptomic analyses revealed extensive reprogramming of the flavonoid pathway in the mutant. Results: Metabolite profiling identified 233 differentially accumulated metabolites (DAMs), with a drastic reduction in anthocyanins, particularly cyanidin- and peonidin-derivatives, together with altered levels of flavonols and flavonoid glycosides. Transcriptome analysis detected 4036 differentially expressed genes (DEGs), with key anthocyanin biosynthetic genes (*DFR*, *ANS*, *UFGT*, *GST*) and *MYB* significantly downregulated. Multi-omics integration confirmed consistent enrichment of flavonoid-related pathways, while correlation network analysis highlighted strong associations between MYB regulators, structural genes, and anthocyanin-type metabolites. Conclusions: Collectively, these findings demonstrate that suppression of a MYB-centered regulatory module underlies the loss of pigmentation in the bud mutant, providing new insights into the molecular regulation of grape skin coloration and a theoretical basis for grape breeding and quality improvement.

## 1. Introduction

Fruit coloration is one of the most important traits determining the commercial value, consumer preference, and nutritional quality of horticultural crops [1,2]. In addition to providing visual attractiveness and marketability, fruit color is closely associated with the accumulation of health-promoting phytochemicals, particularly anthocyanins, which exhibit strong antioxidant, anti-inflammatory, and cardioprotective activities [3,4]. Grapes (*Vitis vinifera* L.) represent one of the most widely cultivated fruit crops worldwide, and the color of grape berries is a key determinant of both fresh fruit quality and wine characteristics. In grape skins, anthocyanins are the primary pigments responsible for red to black coloration [5], and their abundance and composition directly influence the economic and aesthetic value of the fruit as well as its potential health benefits.

Bud sport, a naturally occurring somatic mutation, is a widespread phenomenon in fruit trees and has long been exploited in horticultural breeding to generate phenotypic diversity [6]. Numerous high-value cultivars of apple, citrus, pear, and grape have been derived from bud mutations, which often alter traits such as fruit color, size, ripening time, and seed development [7,8,9]. In grapevine, bud sports represent an especially important source of clonal variation, and several widely cultivated table and wine grape varieties, such as Kyoho, Pinot, and Chardonnay, have originated from somatic mutations affecting berry color, cluster architecture, or ripening behavior [10]. The cultivar Zaoheibao, developed by the Pomology Institute of Shanxi Agricultural University through hybridization of ‘Guibao’ (female parent) and ‘Zaomeigui’ (male parent), followed by colchicine-induced mutagenesis, was officially approved as a tetraploid Eurasian grape variety in 2001 [11]. Zaoheibao has since become one of the dominant grape cultivars for protected cultivation in Shanxi Province. In 2021, a bud sport branch was discovered on a 15-year-old Zaoheibao vine, producing berries with a stable yellow-green skin color instead of the typical purple-black pigmentation. Following graft propagation, this phenotype remained heritable and stable, confirming the occurrence of a true somatic mutation. The striking difference in berry coloration between the wild type and its mutant strongly suggests a disruption in anthocyanin biosynthesis and provides an ideal model to investigate the molecular mechanisms underlying grape skin color variation.

Anthocyanins in grape skins are synthesized via the phenylpropanoid–flavonoid pathway, which involves successive catalytic steps mediated by structural genes [12]. The pathway is initiated by phenylalanine ammonia-lyase (PAL), cinnamate-4-hydroxylase (C4H), and 4-coumarate-CoA ligase (4CL), followed by branch-point enzymes such as chalcone synthase (CHS) and chalcone isomerase (CHI). Downstream reactions involve flavanone 3-hydroxylase (F3H), flavonoid 3′-hydroxylase (F3′H), dihydroflavonol reductase (DFR), and leucoanthocyanidin dioxygenase/anthocyanidin synthase (LDOX/ANS), culminating in anthocyanidin formation [13]. Stabilization occurs through glycosylation by UDP-glucose: flavonoid 3-O-glucosyltransferase (UFGT), while transport into vacuoles requires glutathione S-transferase (GST), ATP-binding cassette (ABCC), and multidrug and toxic compound extrusion (MATE) proteins [14,15]. The expression of these structural genes is largely regulated by the MBW transcriptional complex, consisting of R2R3-MYB, basic helix–loop–helix (bHLH), and WD40 proteins [16,17]. In particular, MYBA1 and MYBA2 are central activators of anthocyanin biosynthesis in grapevine, and their loss-of-function mutations are responsible for the absence of pigmentation in white cultivars [18,19]. Together with partners bHLH and WD40 proteins, they ensure spatiotemporal regulation of anthocyanin accumulation. Disruption of either structural genes or MBW regulators often leads to dramatic alterations in berry coloration. Previous studies have highlighted the pivotal role of R2R3-MYB transcription factors in regulating grape berry coloration [13,20]. For example, MYB regulators such as MYB5a, MYB5b, MYB114, and MYBPA1 have been implicated in the broader control of flavonoid metabolism [21,22,23], thereby influencing the balance between anthocyanins, proanthocyanidins, and flavonols.

In recent years, integrated multi-omics approaches combining metabolomics and transcriptomics have been successfully applied to uncover the molecular mechanisms underlying fruit coloration in grapevine and other fruit species [24,25,26]. For instance, such strategies have revealed the coordinated regulation of structural genes and anthocyanin accumulation in grape cultivars with contrasting skin colors, and similar frameworks have been applied in apple, pear, grape, and pulp to link metabolite variation with transcriptional regulation [24,25,26,27,28]. However, despite these advances, the molecular basis underlying the loss of pigmentation in the bud mutant of Zaoheibao remains unclear. In particular, the integrated relationship between transcriptional regulation and metabolite accumulation in this natural mutant has not been systematically characterized, leaving a significant gap in our understanding of grape skin color variation. This study aimed to clarify the molecular basis of skin color variation between the wild type (WT, purple-black) and bud mutant (MT, yellow-green) of Zaoheibao. By combining transcriptomic and metabolomic analyses, we identified key DEGs and DAMs related to anthocyanin biosynthesis. In particular, we focused on MYB transcription factors and structural genes, and their associations with anthocyanin-type metabolites. These findings provide new insights into the mechanisms of grape skin color formation and contribute to the genetic improvement of grape coloration in grape breeding.

## 2. Materials and Methods

### 2.1. Plant Materials

The grape cultivar Zaoheibao wild type (WT) and its bud mutant (MT) were collected in 2023 at the ripening stage from the experimental vineyard of the Pomology Institute, Shanxi Agricultural University (Taigu County, Jinzhong City, Shanxi Province, China). For each genotype, 15 fruits were collected per biological replicate, with three independent replicates in total. The grape skins (peels) were excised from freshly harvested grapes, immediately frozen in liquid nitrogen, and stored at −80 °C until further use. Three independent biological replicates were prepared for each genotype and subsequently subjected to transcriptome sequencing and LC–MS-based metabolomic analysis. A total of six paired biological samples (3 WT and 3 MT) were used for both metabolomic and transcriptomic sequencing.

### 2.2. Metabolite Profiling and Data Analysis

Metabolite profiling was performed by Biomarker Technologies Co., Ltd. (Beijing, China). Briefly, metabolites were extracted from grape skins and analyzed using a UPLC–ESI–MS/MS system (UPLC: Waters Acquity I-Class PLUS, Waters Corporation, Milford, MA, USA; MS: Applied Biosystems QTRAP 6500+, AB Sciex, Framingham, MA, USA). Chromatographic separation was achieved on a Waters HSS-T3 column (1.8 μm, 2.1 × 100 mm) with a binary mobile phase consisting of solvent A (water with 0.1% formic acid and 5 mM ammonium acetate) and solvent B (acetonitrile with 0.1% formic acid). A gradient elution program was applied at a flow rate of 0.35 mL/min, with the column oven maintained at 50 °C and an injection volume of 2 μL. Mass spectrometric detection was performed using an electrospray ionization (ESI) source operating in both positive and negative ion modes. The source temperature was 550 °C; ion spray voltage was +5500 V/−4500 V; curtain gas, ion source gas I and II were set to 35, 50, and 55 psi, respectively. Data were acquired in multiple reaction monitoring (MRM) mode with optimized declustering potentials and collision energies for each metabolite. Raw data were processed by normalizing peak areas to total ion signals. Principal component analysis (PCA) and Spearman correlation were used to assess sample reproducibility and quality control. Differentially accumulated metabolites (DAMs) were identified based on fold change (FC > 1), Student’s *t*-test (*p* < 0.05), and variable importance in projection (VIP > 1) from orthogonal partial least squares discriminant analysis (OPLS-DA). Metabolites were annotated and classified using the Kyoto Encyclopedia of Genes and Genomes (KEGG), the Human Metabolome Database (HMDB), and the LIPID MAPS Structure Database (LMSD). KEGG pathway enrichment significance was assessed using a hypergeometric distribution test.

### 2.3. Transcriptome Sequencing and Analysis

RNA sequencing was performed by Biomarker Technologies Co., Ltd. (Beijing, China). Total RNA was extracted from grape skins using a plant RNA extraction kit following the manufacturer’s instructions. RNA quality and integrity were assessed by agarose gel electrophoresis, NanoDrop spectrophotometry, and an Agilent 2100 Bioanalyzer (Agilent Technologies, Santa Clara, CA, USA). Sequencing libraries were constructed using the Illumina TruSeq™ RNA Sample Preparation Kit (Illumina Inc., San Diego, CA, USA) and sequenced on the Illumina NovaSeq 6000 platform (Illumina Inc., San Diego, CA, USA) to generate paired-end reads. Raw reads were processed by removing adapters and low-quality sequences using Trimmomatic. Clean reads were mapped to the *Vitis vinifera* reference genome using HISAT2 (v2.2.1). Gene expression levels were normalized as fragments per kilobase of transcript per million mapped reads (FPKM). Differentially expressed genes (DEGs) were identified using DESeq2 (v1.40.2) with thresholds of |log2FC| ≥ 1 and *p* < 0.05. Functional enrichment analyses, including Gene Ontology (GO) and KEGG pathway enrichment, were performed to annotate the biological roles of DEGs.

### 2.4. Statistical Analysis and Data Visualization

All statistical analyses and data visualization were carried out using R software (v4.3.0) and Python (v3.10). PCA and OPLS-DA were performed using the R package (v1.30.0) to evaluate sample clustering and group discrimination. For the joint PCA, the same six biological samples were represented per omics layer to visualize the consistency of clustering between metabolomic and transcriptomic datasets. Each sample thus appears twice (one metabolomic point and one transcriptomic point), yielding 12 data points in total. Normalized transcriptomic (FPKM) and metabolomic datasets were z-score scaled and concatenated prior to analysis using the FactoMineR package in R, allowing visualization of integrated clustering patterns across both omics layers. Differential metabolite analysis was conducted using fold change (FC), Student’s *t*-test, and VIP values as described above. Pearson correlation coefficients were calculated to assess associations between DEGs and DAMs, and correlation networks were visualized using Cytoscape (v3.9.1). Heatmaps of gene expression and metabolite abundance were generated using the R package pheatmap (v1.0.12). Volcano plots, bar charts, bubble plots, and radar charts were constructed with the R package ggplot2 (v3.4.2). All figures were finalized in Adobe Illustrator 2024 (Adobe Systems Inc., San Jose, CA, USA). Unless otherwise stated, results are expressed as mean ± standard deviation (SD), and statistical significance was determined at *p* < 0.05.

## 3. Results

### 3.1. Phenotypic and Metabolomic Separation Between WT and MT Zaoheibao Grape Peels

**Figure 1:**
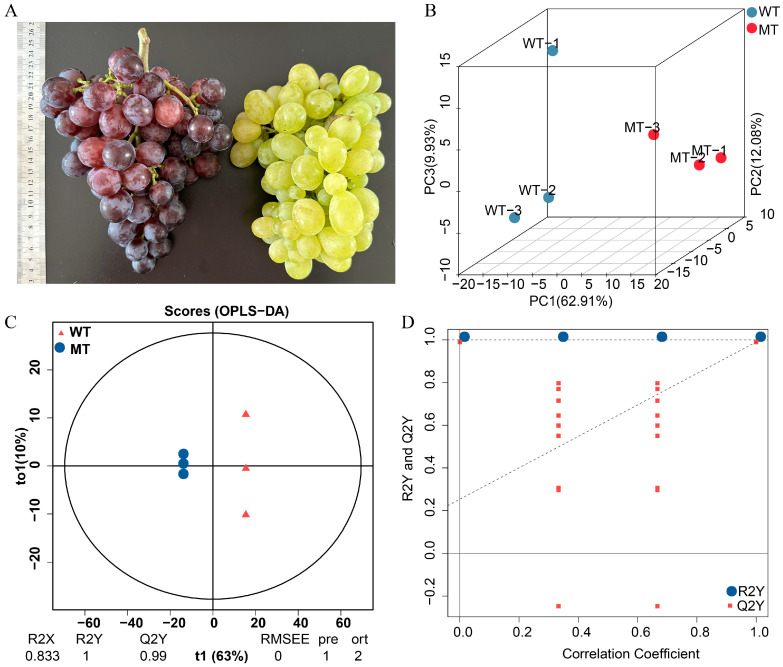
Phenotypic and metabolomic separation between WT and MT Zaoheibao grape peels at maturity. (**A**) Representative fruits showing purple-black peel in WT and yellow-green peel in MT. (**B**) 3D-PCA score plot of metabolomic data. (**C**) OPLS-DA score plot distinguishing WT and MT samples. (**D**) Permutation test of the OPLS-DA model. A positive slope of the Q^2^ regression line and higher R^2^Y than Q^2^ values indicate a valid and robust model.

At maturity, clear phenotypic differences were observed between WT and MT Zaoheibao fruits, with WT peels displaying a purple-black coloration and MT peels exhibiting a yellow-green appearance (Figure 1A). PCA revealed distinct clustering of WT and MT samples, with the first three principal components (PC1, PC2, and PC3) explaining 62.91%, 12.08%, and 9.93% of the total variance, respectively (Figure 1B). The OPLS-DA model further emphasized the group separation, with WT and MT samples clearly distinguished along the predictive component (t1, 63%) (Figure 1C). The model showed excellent explanatory and predictive performance (R^2^X = 0.833, R^2^Y = 1.000, Q^2^ = 0.990). The robustness of the model was confirmed by the permutation test, in which the Q^2^ regression line had a positive slope and all permuted Q^2^ values were lower than the original, while R^2^Y values consistently exceeded Q^2^ values (Figure 1D). Together, these analyses demonstrate that WT and MT peels possess distinct metabolic profiles, consistent with their contrasting pigmentation phenotypes.

### 3.2. Differential Metabolite Analysis Between WT and MT Peels

**Figure 2:**
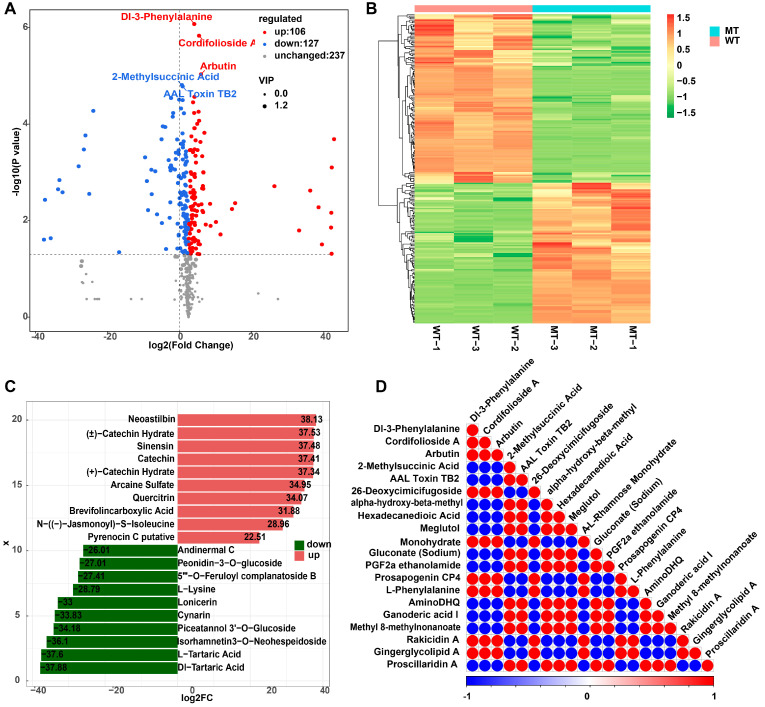
Differential metabolite profiles between WT and MT Zaoheibao peels at maturity. (**A**) Volcano plot showing significantly up-regulated (red) and down-regulated (blue) metabolites (VIP > 1, |log_2_FC| > 1, *p* < 0.05). (**B**) Hierarchical clustering heatmap of all differential metabolites, with samples clearly separated into WT and MT groups. (**C**) Top 10 up- and down-regulated metabolites ranked by log_2_FC. Red bars indicate up-regulated, and green bars indicate down-regulated metabolites. (**D**) Correlation matrix of the top 20 differential metabolites ranked by *p*-value. Red indicates positive correlations, and blue indicates negative correlations; only significant correlations are shown (*p* < 0.05).

A total of 470 metabolites were detected in the peel samples (Table S1). Among these, 233 metabolites were identified as significantly different between WT and MT groups (VIP > 1, |log_2_FC| > 1, *p* < 0.05), comprising 106 up-regulated and 127 down-regulated DAMs (Table S2). The volcano plot illustrated the overall distribution of these differential metabolites (Figure 2A). Hierarchical clustering further confirmed that the metabolic profiles of WT and MT peels were clearly distinct, with biological replicates clustering tightly within each group (Figure 2B). The top 10 metabolites with the largest fold changes were visualized, highlighting compounds such as neoastilbin, catechin hydrate, sinensetin, and arbutin among the up-regulated metabolites, and peonidin-3-O-glucoside, andrimid C, indole-3-carboxaldehyde, and tartronic acid among the down-regulated metabolites (Figure 2C). Correlation analysis of the top 20 differential metabolites showed strong positive and negative associations, suggesting coordinated regulation of several metabolite subsets (Figure 2D). Together, these results indicate that the MT peel exhibits profound metabolic reprogramming relative to WT, with particularly pronounced shifts in flavonoid- and anthocyanin-related metabolites, consistent with the altered pigmentation phenotype.

### 3.3. KEGG Pathway Enrichment Analysis of DAM

**Figure 3:**
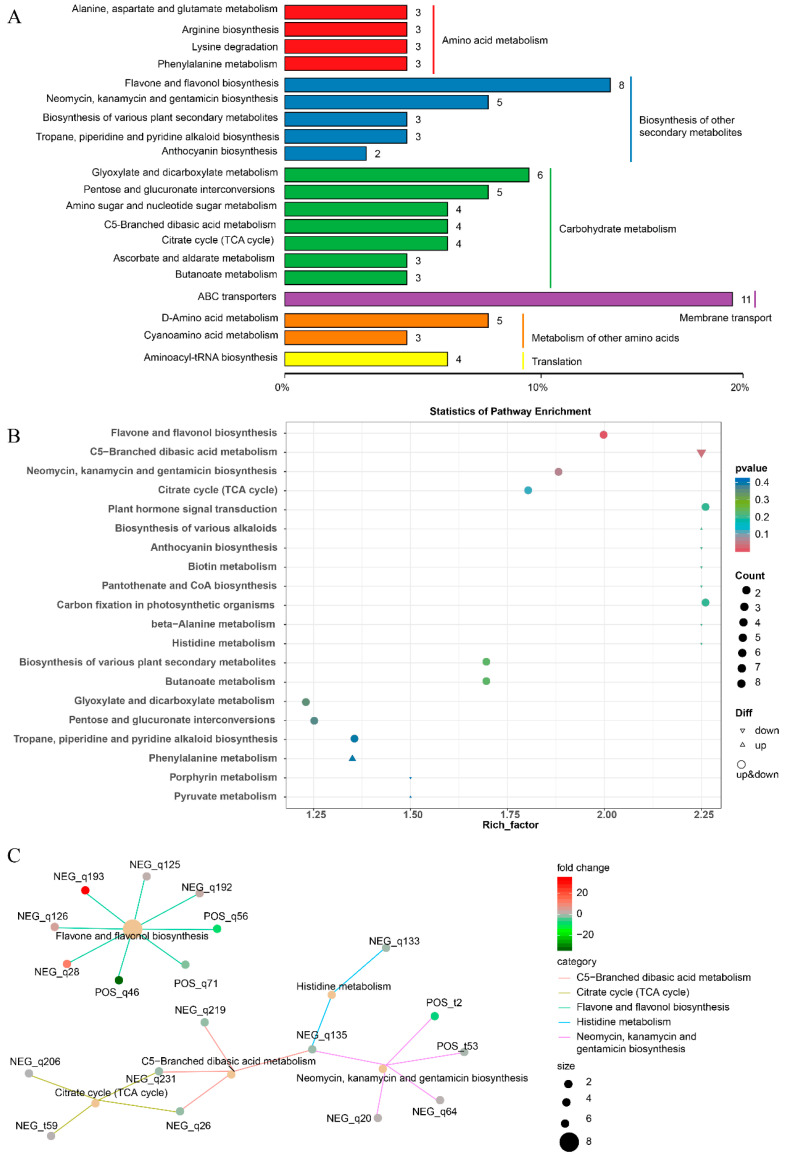
KEGG pathway classification and enrichment of differential metabolites between WT and MT Zaoheibao peels. (**A**) KEGG pathway classification of DAMs. The top 20 pathways with the largest number of annotated differential metabolites are shown; bar length represents metabolite count, and colors indicate KEGG level-2 categories. (**B**) KEGG enrichment bubble plot. The *x*-axis represents the rich factor, the *y*-axis indicates pathway names, bubble size corresponds to the number of enriched metabolites, and color denotes *p*-value. Shapes indicate the regulation pattern of metabolites (up, down, or mixed). (**C**) KEGG enrichment network plot showing representative pathways (≤5) and their associated metabolites. Yellow nodes represent pathways, small colored nodes represent metabolites, node size reflects metabolite count, and color intensity indicates log_2_ fold change.

To further investigate the functional categories of differential metabolites, KEGG annotation and enrichment analyses were performed (Figure 3). A total of 233 differential metabolites (Table S2) were mapped to KEGG pathways, which were mainly classified into amino acid metabolism, carbohydrate metabolism, membrane transport, and secondary metabolite biosynthesis (Figure 3A). Notably, several key pathways related to pigmentation were highlighted, including flavone and flavonol biosynthesis (ko00941) and anthocyanin biosynthesis (ko00942). Enrichment analysis revealed that differential metabolites were significantly enriched in flavone and flavonol biosynthesis, anthocyanin biosynthesis, citrate cycle (ko00020), and C5-branched dibasic acid metabolism (ko00660) (Figure 3B). The bubble plot indicated that these pathways exhibited high rich factors and statistical significance, suggesting that they represent the most impacted metabolic routes. Network visualization further illustrated the associations between representative pathways and their annotated metabolites (Figure 3C). For example, metabolites involved in flavone and flavonol biosynthesis were predominantly down-regulated in MT, consistent with the observed loss of pigmentation. These results demonstrate that the metabolic reprogramming in MT peels is strongly associated with the suppression of flavonoid and anthocyanin biosynthetic pathways, thereby providing a mechanistic explanation for the yellow-green phenotype.

### 3.4. Transcriptomic Analysis of DEGs

**Figure 4:**
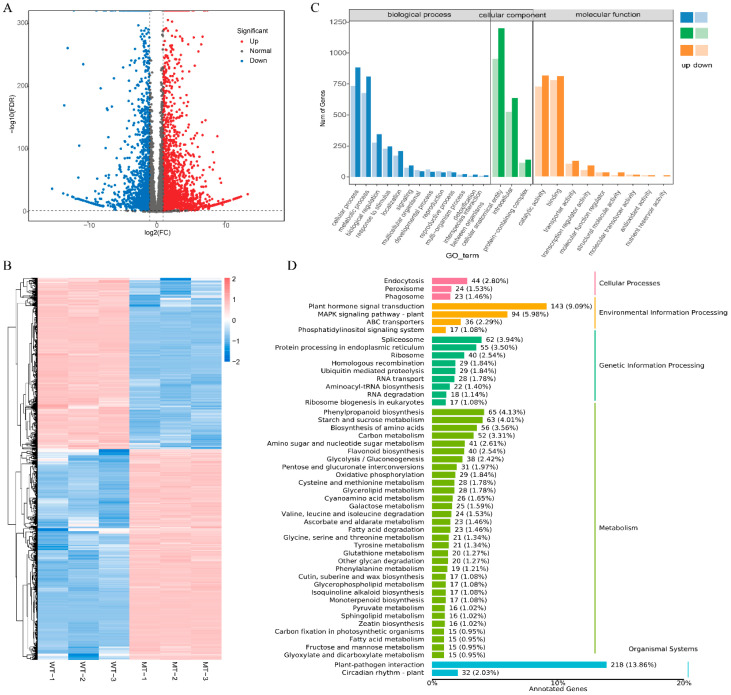
Transcriptomic analysis of WT and MT Zaoheibao peels at maturity. (**A**) Volcano plot of DEGs. Red dots indicate up-regulated genes, blue dots indicate down-regulated genes, and gray dots indicate non-significant genes. (**B**) Hierarchical clustering heatmap of DEGs showing clear separation between WT and MT groups. (**C**) GO enrichment of DEGs categorized into biological process, cellular component, and molecular function. Bar length represents the number of annotated genes. (**D**) KEGG pathway annotation of DEGs. Bars represent pathway categories with corresponding gene counts.

To explore transcriptional reprogramming associated with the altered pigmentation phenotype, RNA-seq analysis was performed on WT and MT Zaoheibao peels. A total of 4036 genes were identified as DEGs, including 2226 up-regulated and 1810 down-regulated genes in MT compared with WT (Table S3). The volcano plot illustrated the overall distribution of DEGs (Figure 4A), while hierarchical clustering revealed that WT and MT samples clustered into two distinct groups, confirming the reproducibility of transcriptomic variation between the genotypes (Figure 4B). GO enrichment analysis showed that DEGs were significantly enriched in multiple categories, including metabolic process, cellular process, and catalytic activity, with notable representation in cellular component organization and membrane-associated functions (Figure 4C). KEGG pathway annotation further revealed that many DEGs were involved in pathways related to flavonoid biosynthesis (ko00941), anthocyanin biosynthesis (ko00942), phenylpropanoid biosynthesis (ko00940), and other metabolic processes, in addition to signal transduction and genetic information processing pathways (Figure 4D). These findings indicate that the peel color variation between WT and MT is underpinned by extensive transcriptional changes, particularly in secondary metabolism pathways relevant to anthocyanin accumulation.

### 3.5. Integrated Transcriptome and Metabolome Analysis

**Figure 5:**
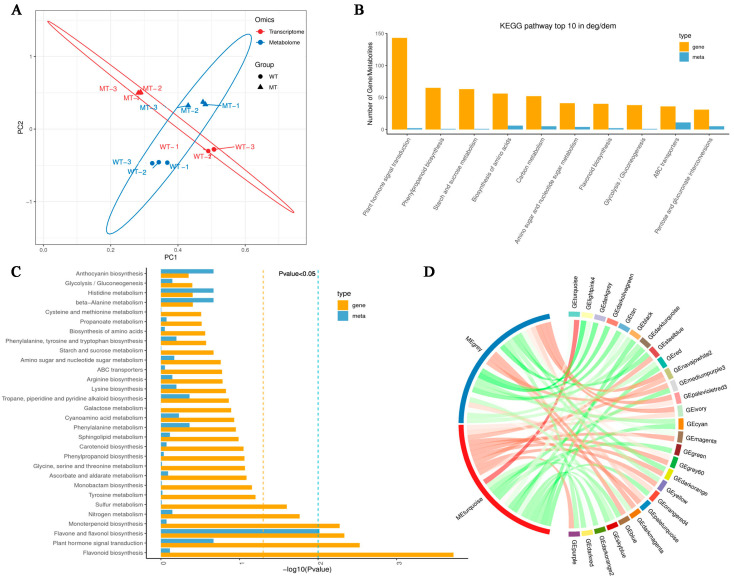
Integrated transcriptomic and metabolomic analysis of WT and MT Zaoheibao peels. (**A**) Joint PCA of transcriptome and metabolome datasets. Each biological sample contributes the transcriptome (red) and the metabolome (blue). Circles and triangles indicate different groups. (**B**) Bar plot showing KEGG pathway annotation counts of the top 10 KEGG pathways with the highest number of DEGs and DAMs. Yellow bars represent DEGs and blue bars represent DAMs. (**C**) KEGG enrichment over-representation analysis of the top 30 significantly enriched pathways shared by DEGs and DAMs. Bar length indicates enrichment significance (−log10 *p*-value). (**D**) Circos plot of weighted gene WGCNA. The left semicircle represents metabolite modules, and the right semicircle represents gene modules. Each ribbon indicates a significant correlation between modules; red = positive correlation and green = negative correlation.

To better elucidate the relationship between transcriptional regulation and metabolic reprogramming underlying peel color variation, we performed an integrated analysis of transcriptome and metabolome datasets (Figure 5). Joint PCA clearly separated WT and MT samples, with transcriptomic (red) and metabolomic (blue) data clustering consistently within each genotype (Figure 5A). This distinct grouping pattern indicates strong coordination between transcriptional and metabolic profiles, suggesting that the differences in gene expression and metabolite accumulation are tightly linked and collectively contribute to the observed peel color divergence between WT and MT. Comparison of KEGG annotation counts revealed that DEGs and DAMs were both highly enriched in several key pathways (Figure 5B). The concurrent presence of both feature types in phenylpropanoid and flavonoid biosynthesis, together with plant hormone signaling and carbohydrate metabolism, suggests coordinated transcriptional and metabolic involvement in peel coloration. Further KEGG enrichment analysis of the top 30 pathways (Figure 5C) emphasized the strong association of differential features with anthocyanin biosynthesis, flavone and flavonol biosynthesis, and phenylpropanoid biosynthesis, which are directly linked to pigment accumulation. The overlap of DEGs and DAMs in key pathways such as phenylpropanoid and flavonoid biosynthesis demonstrates that transcriptional and metabolic reprogramming are closely coupled during color formation. Weighted gene co-expression network analysis (WGCNA) was subsequently used to examine cross-omics associations. The Circos plot showed that metabolite modules enriched in anthocyanins were strongly correlated with gene modules containing UFGT, GST4, and MYBA transcription factors (Figure 5D). These positive correlations suggest that suppression of both structural genes and regulatory factors in MT contributes directly to reduced anthocyanin accumulation. Together, these integrated analyses demonstrate that transcriptional down-regulation of anthocyanin-related genes is tightly coupled with metabolic depletion of anthocyanins, providing a mechanistic explanation for the yellow-green peel phenotype of the MT.

### 3.6. Integrated Correlation Network Between DEGs and DAMs

**Figure 6:**
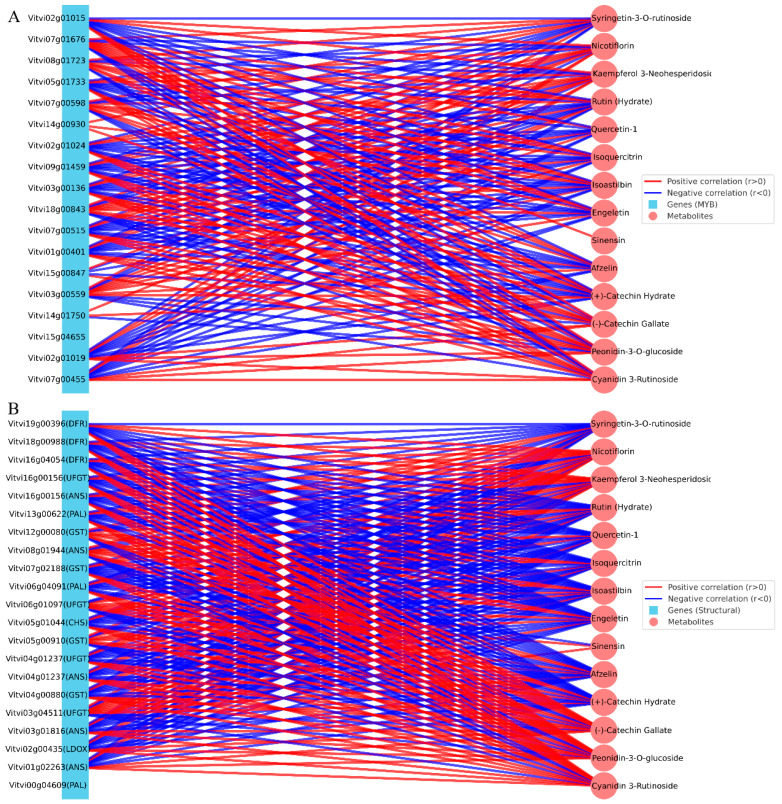
Correlation networks between DEGs and (**A**) network of selected MYB transcription factors and flavonoid-related DAMs. (**B**) Network of selected structural genes (top 5 DEGs from key anthocyanin biosynthesis and transport steps) and flavonoid-related DAMs. Edges represent significant Pearson correlations (|r| ≥ 0.8, *p* < 0.01), with red indicating positive and blue indicating negative correlations. Squares denote genes and circles denote metabolites.

To further elucidate the molecular basis underlying the altered grape coloration of the bud mutant, a correlation network was constructed by integrating transcriptome and metabolome data (Figure 6). Based on the annotation information, we first screened 18 differentially expressed MYB transcription factors including Vvi07g00515 (MYBA1), Vvi15g00465 (MYBA2), Vvi02g01019 (MYBPA1), Vvi07g00455 (MYB5a), and Vvi15g00847 (MYB5b) (Figure 6A). These TFs are well-known regulators of anthocyanin biosynthesis. We selected 21 structural genes directly involved in pigment biosynthesis and transport, also based on |log2FC| ranking (top 5 per category). These included Vvi19g00396 (DFR, dihydroflavonol reductase), Vvi16g00156 (ANS/LDOX, anthocyanidin synthase), Vvi16g00150 (UFGT, UDP-glucose:flavonoid 3-O-glucosyltransferase), Vvi05g01917 (CHI, chalcone isomerase), and Vvi08g01948 (GST, glutathione S-transferase) (Figure 6B). All these genes represent key steps of the phenylpropanoid–flavonoid–anthocyanin pathway, from precursor biosynthesis to anthocyanin glycosylation and vacuolar sequestration. Pearson correlation analysis (|r| ≥ 0.8, *p* < 0.01) revealed strong associations between these DEGs and 14 DAMs related to flavonoid metabolism. Among them, Cyanidin 3-rutinoside and Peonidin-3-O-glucoside were classified as anthocyanins, whereas (−)-Catechin gallate and (+)-Catechin hydrate belonged to flavanols. In addition, several metabolites were grouped into flavonoid glycosides, such as Afzelin and Sinensin, while the majority were flavonols, including Engeletin, Isoastilbin, Isoquercitrin, Quercetin-1, Rutin (hydrate), Kaempferol 3-neohesperidoside, Nicotiflorin, and Syringetin-3-O-rutinoside (Figure 6). Taken together, these results highlighted a coherent gene–metabolite correlation module, in which *MYB* and their downstream structural genes (*DFR*, *ANS*, *UFGT*, *CHI*, *GST*) were tightly linked with anthocyanin-type DAMs, especially cyanidin- and peonidin-derivatives. This suggests that transcriptional reprogramming of the MBW regulatory complex and its structural targets underlies the dramatic reduction of anthocyanin accumulation in the bud mutant berries, thereby explaining the color transition from purple-black to yellow-green.

## 4. Discussion

Bud sport is a common somatic mutation in fruit trees and serves as an important source of phenotypic diversity in horticultural crops [6]. The bud mutant of Zaoheibao exhibited a remarkable shift in grape skin color, changing from the typical purple-black pigmentation of the WT to a stable yellow-green phenotype (Figure 1A). Since anthocyanins are the primary pigments responsible for red to black coloration in grape skins, the absence of pigmentation in the mutant strongly suggests a disruption of anthocyanin biosynthesis and accumulation. Such color variation is not only critical for fruit appearance and market value but also impacts the nutritional and antioxidant properties of berries [29]. Similar cases of skin color alteration have been reported in grapevine bud sports such as Pinot and Chardonnay, where impaired anthocyanin accumulation resulted in distinctive clonal variants [30,31]. These findings emphasize the central role of anthocyanins in determining grape color and highlight bud mutations as valuable natural materials for dissecting the molecular mechanisms underlying pigmentation.

Anthocyanins are synthesized via the phenylpropanoid–flavonoid pathway, which encompasses a wide array of metabolites including flavanols, flavonols, and flavonoid glycosides, in addition to anthocyanins themselves [32]. Metabolomic comparisons revealed that the most conspicuous metabolic change between WT and MT skins was the drastic reduction of anthocyanins, particularly cyanidin-3-rutinoside and peonidin-3-O-glucoside (Figure 2 and Figure 3). These compounds are the dominant pigments in most red- and black-skinned grapes, and their loss is consistent with the observed yellow-green phenotype. Previous studies have shown that the absence of anthocyanins is a hallmark of white grape cultivars, which often results from impaired MYBA regulation of anthocyanin biosynthetic genes [33,34]. The parallel between our bud mutant and naturally white grapes suggests that similar regulatory defects may underlie the color transition in Zaoheibao MT. In addition to anthocyanins, we observed significant shifts in other flavonoid subclasses, including flavonols (rutin, isoquercitrin, and syringetin derivatives) and flavanols (catechins). These metabolites not only contribute to grape color co-pigmentation but also play important roles in photoprotection and antioxidant defense [35]. The increased relative abundance of flavonols in the mutant may represent a compensatory metabolic adjustment when anthocyanin biosynthesis is blocked. This metabolic compensation likely arises from the diversion of dihydroflavonol precursors toward the flavonol branch when the anthocyanin pathway is suppressed. Such metabolic re-routing has been reported in other fruit crops, where suppression of anthocyanin accumulation leads to the redirection of precursors toward alternative flavonoid branches [36]. The enrichment of phenylpropanoid and flavonoid biosynthesis pathways in KEGG analyses further highlights the central role of these metabolic routes in mediating the phenotypic divergence. Together, these results suggest that the color loss in Zaoheibao MT is not a simple absence of anthocyanins but reflects a broader remodeling of flavonoid metabolism, with consequences for both pigmentation and potential nutritional value.

The transcriptome analysis revealed that multiple structural genes of the anthocyanin pathway were markedly downregulated in the mutant skins (Figure 4, Table S3). Representative structural genes, including *DFR* (*Vvi19g00396*), *ANS/LDOX* (*Vvi16g00156*), and *UFGT* (*Vvi16g00150*), which are indispensable for the late steps of anthocyanin biosynthesis and stabilization. In addition, *GST* (*Vvi08g01948*), a key transporter mediating vacuolar sequestration of anthocyanins, was also suppressed. Such coordinated downregulation is consistent with the observed loss of pigmentation [37]. Equally important was the reduced expression of transcriptional regulators (*MYBA1*, *Vvi07g00515*; *MYBA2*, *Vvi15g00465*), which have been widely recognized as central regulators of grape coloration. Similar phenomena have been reported in other species: *MdMYB1* in apple [38], *PpMYB10* in peach [39], and *LcMYB1* in litchi [40], all of which control anthocyanin biosynthetic genes and determine skin or flesh pigmentation. These parallels suggest that the bud mutation in Zaoheibao disrupts a conserved MYB-centered regulatory module, leading to the failure of anthocyanin accumulation in mutant berries. Although MYB-dependent regulation appears central, other transcriptional and signaling factors (*bHLH*, *WD40*, and hormone-related genes) may also contribute to the observed transcriptional–metabolic reprogramming.

Integrative analysis of transcriptomic and metabolomic datasets provided a coherent view of the mechanisms underlying the skin color transition in Zaoheibao (Figure 5). Both DEGs and DAMs were significantly enriched in the flavonoid and anthocyanin biosynthesis pathways, confirming that the bud mutation primarily affects pigment-related metabolism. Similar strategies have been successfully applied in other fruit crops, including apple, peach, and litchi, to link transcriptional regulation with pigment accumulation, highlighting the generality of this framework across species [38,39,40]. Correlation network analysis further revealed the close associations between regulatory genes and metabolite variation (Figure 6). MYB transcription factors were strongly correlated with anthocyanin-type DAMs, including cyanidin-3-rutinoside and peonidin-3-O-glucoside, reinforcing their pivotal roles as activators of the anthocyanin pathway. Structural genes such as *DFR*, *ANS*, *UFGT*, *CHI*, and *GST* also exhibited tight correlations with multiple flavonoid subclasses, suggesting that the loss of pigmentation in the mutant was accompanied by broad metabolic re-routing within the flavonoid network [41]. Notably, correlations extended beyond anthocyanins to flavonols and flavonoid glycosides, indicating that perturbation of MYB-mediated regulation reshapes the entire flavonoid metabolic landscape [42]. Taken together, these integrated analyses provide compelling evidence that the yellow-green phenotype of the Zaoheibao bud mutant is the result of a disrupted transcriptional–metabolic module, in which suppression of MYB regulators leads to downregulation of structural genes and subsequent depletion of anthocyanins. The correlation-based networks not only recapitulate known regulatory relationships but also highlight candidate genes and metabolites for future functional validation.

## 5. Conclusions

This study combined transcriptomic and metabolomic analyses to investigate the molecular basis of berry skin color variation in the grape cultivar Zaoheibao and its bud mutant. The mutant exhibited a stable yellow-green phenotype associated with a drastic reduction in anthocyanin accumulation. Integrated analyses revealed that key anthocyanin biosynthetic genes (*DFR*, *ANS*, *UFGT*, *GST*) and regulatory factors *MYB* were markedly downregulated, leading to impaired pigment biosynthesis and vacuolar sequestration. Correlation network analysis further confirmed tight associations between these genes and anthocyanin-type metabolites. Collectively, our findings demonstrate that transcriptional suppression of the MYB-centered regulatory module disrupts anthocyanin biosynthesis, thereby driving the loss of pigmentation in the bud mutant. This work provides new insights into the molecular mechanisms underlying grape skin coloration and offers a reference for grape breeding targeting berry color improvement.

## Data Availability

The article contains all raw data. For further inquiries, please contact the corresponding author.

## Supplementary Materials

The following supporting information can be downloaded at: https://www.mdpi.com/article/10.3390/metabo15100675/s1, Table S1: Detected metabolites in the peels of WT and MT of *Vitis vinifera* Zaoheibao; Table S2: DAMs identified between WT and MT peels of *Vitis vinifera* Zaoheibao; Table S3: DEGs identified between WT and MT peels of *Vitis vinifera* Zaoheibao.

## Abbreviations

The following abbreviations are used in this manuscript:

ANS/LDOX	Anthocyanidin synthase/Leucoanthocyanidin dioxygenase
bHLH	Basic helix–loop–helix (transcription factor)
CHI	Chalcone isomerase
CHS	Chalcone synthase
C4H	Cinnamate-4-hydroxylase
DAMs	Differentially accumulated metabolites
DEGs	Differentially expressed genes
DFR	Dihydroflavonol reductase
F3H	Flavanone 3-hydroxylase
F3′H	Flavonoid 3′-hydroxylase
FPKM	Fragments per kilobase of transcript per million mapped reads
GST	Glutathione S-transferase
HMDB	Human Metabolome Database
KEGG	Kyoto Encyclopedia of Genes and Genomes
LC–MS	Liquid chromatography–mass spectrometry
LMSD	LIPID MAPS Structure Database
MBW	MYB–bHLH–WD40 transcriptional complex
MRM	Multiple reaction monitoring
MT	Mutant (bud sport of *Vitis vinifera* ‘Zaoheibao’)
OPLS-DA	Orthogonal partial least squares discriminant analysis
PAL	Phenylalanine ammonia-lyase
PCA	Principal component analysis
PC	Principal component
QC	Quality control
RNA-seq	RNA sequencing
UFGT	UDP-glucose: flavonoid 3-O-glucosyltransferase
VIP	Variable importance in projection
WT	Wild type (*Vitis vinifera* ‘Zaoheibao’)
WGCNA	Weighted gene co-expression network analysis
